# Exploring New Characteristics: Using Deep Learning and 3D Reconstruction to Compare the Original COVID-19 and Its Delta Variant Based on Chest CT

**DOI:** 10.3389/fmolb.2022.836862

**Published:** 2022-03-11

**Authors:** Na Bai, Ruikai Lin, Zhiwei Wang, Shengyan Cai, Jianliang Huang, Zhongrui Su, Yuanzhen Yao, Fang Wen, Han Li, Yuxin Huang, Yi Zhao, Tao Xia, Mingsheng Lei, Weizhen Yang, Zhaowen Qiu

**Affiliations:** ^1^ College of Information and Computer Engineering, Northeast Forestry University, Harbin, China; ^2^ China United Network Communications Corporation Heilongjiang Branch, Harbin, China; ^3^ Hongqi Hospital Affiliated to Mudanjiang Medical University, Mudanjiang, China; ^4^ Zhangjiajie Hospital Affiliated to Hunan Normal University, Zhangjiajie, China; ^5^ Medical College of Jishou University, Jishou, China; ^6^ Heilongjiang Tuomeng Technology Co. Ltd., Harbin, China

**Keywords:** Original COVID-19, Delta Variant, chest CT, deep learning, three-dimensional reconstruction, stereoscopic segmentation, quantitative analysis

## Abstract

**Purpose:** Computer-aided diagnostic methods were used to compare the characteristics of the Original COVID-19 and its Delta Variant.

**Methods:** This was a retrospective study. A deep learning segmentation model was applied to segment lungs and infections in CT. Three-dimensional (3D) reconstruction was used to create 3D models of the patient’s lungs and infections. A stereoscopic segmentation method was proposed, which can subdivide the 3D lung into five lobes and 18 segments. An expert-based CT scoring system was improved and artificial intelligence was used to automatically score instead of visual score. Non-linear regression and quantitative analysis were used to analyze the dynamic changes in the percentages of infection (POI).

**Results:** The POI in the five lung lobes of all patients were calculated and converted into CT scores. The CT scores of Original COVID-19 patients and Delta Variant patients since the onset of initial symptoms were fitted over time, respectively. The peak was found to occur on day 11 in Original COVID-19 patients and on day 15 in Delta Variant patients. The time course of lung changes in CT of Delta Variant patients was redetermined as early stage (0–3 days), progressive and peak stage (4–16 days), and absorption stage (17–42 days). The first RT-PCR negative time in Original COVID-19 patients appeared earlier than in Delta Variant patients (22 [17–30] vs. 39 [31–44], *p* < 0.001). Delta Variant patients had more re-detectable positive RT-PCR test results than Original COVID-19 patients after the first negative RT-PCR time (30.5% vs. 17.1%). In the early stage, CT scores in the right lower lobe were significantly different (Delta Variant vs. Original COVID-19, 0.8 ± 0.6 vs. 1.3 ± 0.6, *p* = 0.039). In the absorption stage, CT scores of the right middle lobes were significantly different (Delta Variant vs. Original COVID-19, 0.6 ± 0.7 vs. 0.3 ± 0.4, *p* = 0.012). The left and the right lower lobes contributed most to lung involvement at any given time.

**Conclusion:** Compared with the Original COVID-19, the Delta Variant has a longer lung change duration, more re-detectable positive RT-PCR test results, different locations of pneumonia, and more lesions in the early stage, and the peak of infection occurred later.

## 1 Introduction

Coronavirus disease 2019 (COVID-19) is a global epidemic caused by severe acute respiratory syndrome coronavirus 2 (SARS-CoV-2) (Qu et al., 2020). It was first found in Wuhan city, China in late 2019. It has the characteristics of rapid transmission and high mortality, which seriously endangers human health. With the persistence of the epidemic and the increase in the number of infected persons, SARS-CoV-2 has continuously evolved and mutated, and a variety of concerned variants such as Alpha, Beta, Gamma, and Delta have been generated in Europe, Africa, South America, and Asia (Koyama et al., 2020). Among them, the Delta variant of concern (Delta VOC, also known as lineage B.1.617.2) has become the world’s dominant strain since it was discovered in India in October 2020 (Choudhary et al., 2021). Its transmission capacity and viral load have upgraded significantly. It also has the characteristics of having a long negative conversion time and easily developing into critical illness (Wang et al., 2021).

We searched papers from Web of Science, Engineering Village, PubMed, and China National Knowledge Infrastructure from the earliest available date to November 30, 2021. We found a large number of studies describing the clinical course of patients with COVID-19 and its various variants, as well as many kinds of literature similar to this study describing the comparison of COVID-19 with H1N1, seasonal influenza, SARS, and MERS (Petrosillo et al., 2020; Yin et al., 2020; Bai and Tao, 2021; Brehm et al., 2021), but almost none of the studies directly compared the Original COVID-19 with the COVID-19 Delta Variant. Currently, gene sequencing is the only mainstream method to identify and distinguish the Delta Variant from the Original COVID-19. In this study, we collected CT scans and clinical data from patients with the Original COVID-19 and Delta Variant. We used deep learning, three-dimensional (3D) reconstruction, and data analysis methods for comparative analysis, aiming to find out the characteristics that distinguish the two in CT and clinical, and summarize some specific manifestations about the Delta Variant to provide references for clinical diagnosis. In addition, we have released an online COVID-19 modeling platform to help alleviate the COVID-19 epidemic worldwide, which includes functions such as COVID-19 lesion detection, 3D reconstruction of lungs and infections, and quantitative calculation of infections.

## 2 Materials and Methods

### 2.1 Materials

#### 2.1.1 Data Collection

This was a retrospective study. A total of 35 patients admitted to Hongqi Hospital Affiliated to Mudanjiang Medical University (from Heilongjiang Province, China) from April 1 to 26 May 2020 were collected to form the Original COVID-19 dataset, including 24 male and 11 female patients. A COVID-19 Delta Variant dataset was collected from 72 patients admitted to Zhangjiajie City People’s Hospital (from Hunan Province, China) from July 28 to August 13, 2021, including 29 male and 43 female patients. All patients were included in this study after RT-PCR confirmation. Neither dataset contained asymptomatic infected persons. The two datasets contained all CT scans of each patient during hospitalization, as well as clinical data such as the onset time of initial symptoms and the first negative date of RT-PCR.

Discharge standards conformed to the Guidelines for the Diagnosis and Treatment of Novel Coronavirus Infection produced by the Chinese National Health Commission (Trial Version eight or earlier versions) (National Health Commission, 2020). The selected cases were divided into the youth (under 45 years), the middle-aged (45–59 years), and the elderly (60–89 years) according to age classification criteria issued by the World Health Organization. This study was approved by all relevant hospital ethics committees. Since the study was retrospective and posed no potential risk to patients, the requirement for informed consent was waived.

#### 2.1.2 Equipment and Parameters

Hongqi Hospital Affiliated to Mudanjiang Medical University used Nms Neuviz 128 layers CT to obtain chest CT of Original COVID-19 patients. Zhangjiajie City People’s Hospital used TOSHIBA Aquilion Lightning CT to obtain chest CT of Delta Variant patients. The tube voltage and the current tube were 120 kVp and 100–200 mA, respectively. All data were reconstructed with 1.0 mm increment. The matrix was 512 × 512 mm^2^. Images were reconstructed using a sharp reconstruction kernel for parenchyma. The lung window level and the lung window width of the Original COVID-19 CT scans were −500 HU and 1600 HU, respectively. The lung window level and the lung window width of the Delta Variant CT scans were −600 HU and 1600 HU, respectively.

### 2.2 Segmentation and Quantification of COVID-19

#### 2.2.1 2.5D Segmentation Model

In the past 5 years, the deep learning segmentation algorithm based on 2.5D idea has shown excellent performance in medical image analysis problems such as liver tumor segmentation, chronic stroke lesion, and pancreas segmentation (Han, 2017; Li et al., 2018; Zhou et al., 2021). Its main idea is to transform 3D problems into multiple 2D problems. For example, using a 2D convolutional neural network to take several adjacent slices of the 3D graph as the input of the model and output a 2D segmentation graph corresponding to the central slice. In previous studies, our team proposed a rapid, accurate, and machine-agnostic 2.5D segmentation algorithm for CT-based COVID-19 diagnosis (Zhou et al., 2020). This model used spatial normalization and signal normalization to unify the resolution, dimension, and each voxel’s signal intensity of CT scans, which enables it to process any CT datasets generated by different CT machines. To solve the problem of data scarcity in deep learning, the model also provided a CT scan simulator to describe the dynamic changes of COVID-19 infection regions. The CT scan simulator can be used not only to increase experimental data, but also to prevent model overfitting. For CT data after normalization, the infected regions are segmented by U-Net (Ronneberger et al., 2015) neural network along with the three orthogonal directions of the coronal section, median sagittal section, transverse section, and then the three-segmented masks are fused to obtain the segmented infected regions. The DICE score is used to evaluate the segmentation model, and its expression is:

$$\mathrm{DICE}=\frac{2\cdot |\left(V_{\mathrm{seg}}\cap V_{\mathrm{gt}}\right)|}{\left|V_{\mathrm{seg}}|+|V_{\mathrm{gt}}\right|}$$ (1)

where *V*_*seg*_ is the predicted segmentation result, *V*_*gt*_ is the ground truth segmentation result, and |*V*_*seg*_| denotes the cardinality of the set *V*_*seg*_.

Since the model has achieved excellent performance on multiple COVID-19 CT datasets after training, this study continues to use it for COVID-19 segmentation.

#### 2.2.2 Infection Volume Calculation

STL (STereoLithography) is a storage format for describing geometric information of 3D objects with triangular mesh. We used STL to store the results of the 2.5D segmentation model. The volume calculation principles and methods of STL are as follows: The spatial area surrounded by all triangular patches of triangular mesh is closed. Specify a projection plane, and calculate the signed volume of a convex pentahedron composed of a projection triangle of triangular patches on a projection plane and the triangular patches. Then, calculate the algebraic sum of the signed volume of all convex pentahedra to get the volume of an STL file (Wang, 2009). By calculating the volume of the STL files, the percentages of infection (POI) in COVID-19 patients can be obtained for quantitative analysis.

#### 2.2.3 COVID-19 Modeling Platform

**FIGURE 1:**
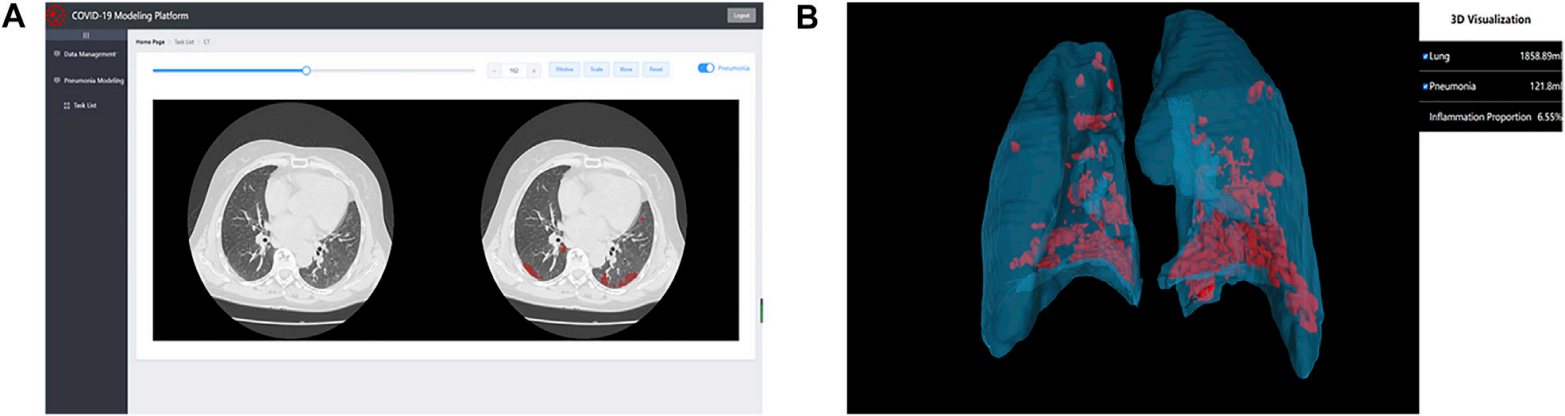
An online COVID-19 modeling platform for COVID-19 diagnosis. **(A)** shows the function of 2D CT reading. **(B)** shows the function of 3D model reading.

Computer-aided diagnosis systems based on artificial intelligence (AI) play a key role in the early identification and diagnosis of suspected and confirmed patients during the COVID-19 pandemic (Dou et al., 2021). Previous CT examination reports can only roughly estimate the range of lesions, which cannot be quantitatively analyzed. It is also difficult to determine whether the lesions change and whether the treatment plan is effective after multiple checks. The 2.5D segmentation model for COVID-19 diagnosis proposed by our team can not only identify the subtle lesions that are difficult to identify by the human eyes, but also improve the accuracy of COVID-19 screening as a parallel method of RT-PCR test, and assist in timely clinical diagnosis. It can also quantitatively analyze the degree of involvement of each lung lobe and lung segment, and continuously observe the prognosis of the disease course. It suggests that patients are in what stage of the disease, which is an important reference for doctors to monitor the progress of the disease and guide clinical decisions. To make this excellent model more widely accepted and used, we developed an online COVID-19 modeling platform (http://111.42.74.202:8000/covid) shown in Figure 1, to facilitate COVID-19 epidemic prevention and control. The front end of the website was written using vue. js, xtk. js, and cornerstone. js, providing a friendly graphical user interface for doctors and researchers. The back end used Flask and MySQL. After uploading a CT scan (DICOM format), the platform can complete a 3D reconstruction of lung and infection in 2 min, and quantify the infection. In addition, the platform also has some useful functions, e.g., 2D CT reading and 3D model reading. During the COVID-19 Delta Variant outbreak in Zhangjiajie city, Hunan Province in July 2021 and Heihe City, Heilongjiang Province in November 2021, the COVID-19 modeling platform was used by doctors to assist in clinical diagnosis, making significant contributions to fight against COVID-19. Accounts of the platform are available on request to doctors and researchers. Five CT scans of COVID-19 patients (DICOM format) have been uploaded to https://github.com/RickieLim/COVID-19-Modeling-Platform as test data.

### 2.3 Stereoscopic Segmentation of Lung Lobes and Segments

The lung can be divided into the left lung and the right lung in morphological structure. The left lung can be subdivided into the left upper lobe and the left lower lobe by the oblique fissure. The right lung can be subdivided into the right upper lobe, the right middle lobe, and the right lower lobe by horizontal fissure and oblique fissure (Fourdrain et al., 2017). According to the direction of the bronchi and pulmonary arteries in the 3D space, the five lung lobes can be subdivided into 18 lung segments. Quantitative analysis of lesion distribution in each lung lobe and lung segment can help understand the dynamic changes of lung infections in patients and update the progress of the disease in time, which is of great significance for clinical diagnosis and treatment of COVID-19 pneumonia. Concretely speaking, (1) calculating the POI value in each region of five lung lobes and 18 lung segments can determine the current degree of infection diffusion in patients; (2) in the early CT taken after the onset of initial symptoms, the frequency of lesions in each lung lobe and lung segment is highly correlated with the severity of the disease. Statistics of the frequency of lesions in each region, the higher the frequency of a region, indicating that the region’s condition will be faster aggravation; and (3) combining POI value and the frequency of lesions in each region, the predilection sites of the disease can be determined.

In order to achieve accurate segmentation of five lung lobes and 18 lung segments, we proposed a computer-aided stereoscopic segmentation algorithm for lung lobes and lung segments based on chest CT, which can quickly and accurately complete the extraction and modeling of lung lobes and segments without trauma. STL files of lung lobes and lung segments can be obtained by using this algorithm. Use Boolean operation between STL of each lung lobe or lung segment and the total infection STL (obtained by the 2.5D segmentation model) to obtain infection STL of each lung lobe and lung segment, and then calculate lesion frequency and POI value in each region.

**FIGURE 2:**
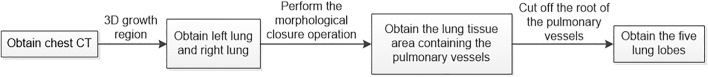
The principle flowchart of lung lobes segmentation.

**FIGURE 3:**
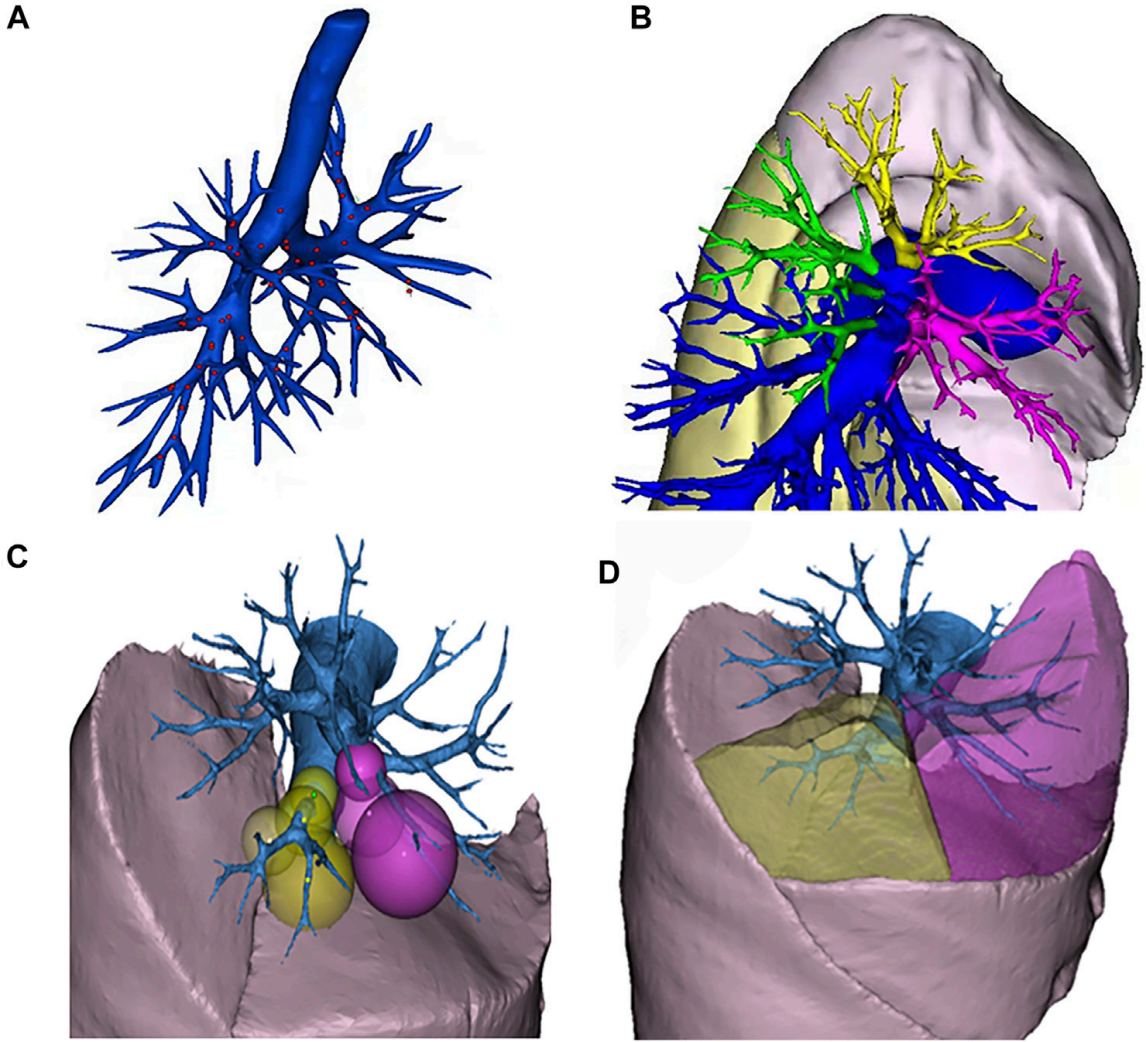
Schematic diagram of segmental lung segmentation. Schematic diagram of segmental lung segmentation. **(A)** shows the initial seed points of the centerline. **(B)** shows the labeling of the pulmonary artery roots root. **(C)** shows the spherical expansion growth. **(D)** shows the formation of lung segments.

The algorithm is divided into two steps. In the first step, the lung is subdivided into five lobes. The principle flowchart of lung lobes segmentation is shown in Figure 2. Firstly, through the analysis of CT raw data, the histograms are obtained. Set the initial segmentation threshold and select the seed point based on this. Taking the seed point as the starting point, lung tissue can be extracted by the 3D growth region, and two unrelated regions are obtained. Label the two as left lung and right lung, respectively. Secondly, since the gray value of pulmonary vessels in chest CT is significantly higher than the gray value of lung tissue, the extracted left lung and right lung only contain fine or low gray value pulmonary vessels. Pulmonary vessels with coarser and higher gray values are not included. The closed operation is performed on the lung tissue area to fill the pulmonary vessels, and obtain the lung tissue area containing the pulmonary vessels. Finally, the root of the pulmonary vessels and the trachea are at the edge of the lung. The connection between the pulmonary vessels at the edge of the lung tissue and the root of the pulmonary vessels can be cut off by corrosion operation to realize the separation of each lung lobe and pulmonary vessels. The obtained five interconnected domains are the five lung lobes. In the second step, further segmentation of the lung based on the generation of the five lung lobes. Multiple pulmonary artery roots are selected in the candidate lung lobe regions for labeling (Figure 3B), and the points on the centerline of the selected pulmonary artery and its smaller branches are set as the initial seed points (Figure 3A). Each seed point expands equidistantly spherically according to a constant rate and 26 neighborhood regions growing rule (Figure 3C). Stop growing when the growing area reaches the lung boundary, horizontal fissure, oblique fissure, or the connection of the two growth zones. The expansion boundary of seed points on each branch of the artery in lung segments forms the regional scope of lung segments (Figure 3D).

### 2.4 Chest CT Evaluation

Kazerooni et al. (1997) proposed a semi-quantitative scoring system for CT in 1997 to evaluate the severity of diseases by quantifying the degree of involvement of each lung lobe. This method required several radiologists with longer qualifications to analyze CT scans and visually score the five lung lobes (ranging from 0 to 5): 0 means no lesion; one indicates that the lesion volume is less than 5% of the lung lobe volume; two indicates that the lesion volume accounts for 5%–25% of the lung lobe volume; three indicates that the lesion volume accounts for 26%–49% of the lung lobe volume; four indicates that the lesion volume accounts for 50%–75% of the lung lobe volume; five indicates that the lesion volume exceeds 75% of the lung lobe volume. The final score of each lobe was the average of the scores proposed by multiple doctors. The total score of each CT scan is the sum of the visual scores of the five lung lobes, ranging from 0 to 25. When doctors had different opinions on the scores of a certain lung lobe, they needed to be reviewed again by more experienced senior doctors. This method has been widely used by scholars in the past 24 years, especially during SARS in 2002 and COVID-19 in 2019. Many highly cited papers studying pneumonia have cited this scoring system. However, this semi-quantitative scoring system dominated by visual scoring has great limitations. It not only requires many experienced radiologists to spend a lot of time reading CT, but also has the risks of large manual error and inaccurate scoring results. We improved this semi-quantitative scoring system and proposed a fully quantitative AI-based scoring system without manual intervention. Boolean operation (intersection operation) is performed on the total infection STL of each CT scan obtained by the 2.5D segmentation model and the STL of each lung lobe obtained by the stereoscopic segmentation method to obtain the infection STL corresponding to each lung lobe. The infection is then calculated for each lobe as a percentage of the lung and mapped to the AI-based scoring system. This can not only improve the accuracy of the scoring system but also evaluate the degree of lung involvement in a very short time, providing a timely reference for clinical diagnosis.

The characteristics of CT were described concerning the recommendations of the Fleischner Society Naming Committee (Hansell et al., 2008). The main features of CT include ground-glass opacity (GGO), crazy-paving pattern (GGO with interlobular and interlobular septal thickening), solidification (substance opaque, lower blood vessels blurred), and linear opacity (rough or curved opacity or fine subpleural reticular tissue disorder).

During the COVID-19 epidemic from January to February 2020, Pan et al. (2020) used the semi-quantitative CT scoring system and regression analysis to divide patients with mild symptoms into four stages: (1) early stage, 0–4 days after the onset of initial symptoms; (2) progressive stage, 5–8 days after the onset of initial symptoms; (3) peak stage, 9–13 days after the onset of the initial symptoms; (4) absorption stage, ≥14 days after the onset of initial symptoms. This conclusion has been widely accepted by the academic community.

### 2.5 Statistical Analysis

Statistical analyses were performed by using software (SPSS 26, IBM). Unless otherwise specified, continuous variables were represented by mean ± standard deviation or median [interquartile range], and categorical variables were represented by counts and percentages. Comparisons of continuous variables were performed with the Mann-Whitney *U* test. Categorical variables were compared using *χ* ^2^ test. The SPSS curve estimation module was used to quantitatively assess the change in total CT score of lung involvement over time. *p*-values less than 0.05 were considered statistically significant.

## 3 Results

### 3.1 3D Reconstruction and Stereoscopic Segmentation of Lung and Infection

**FIGURE 4:**
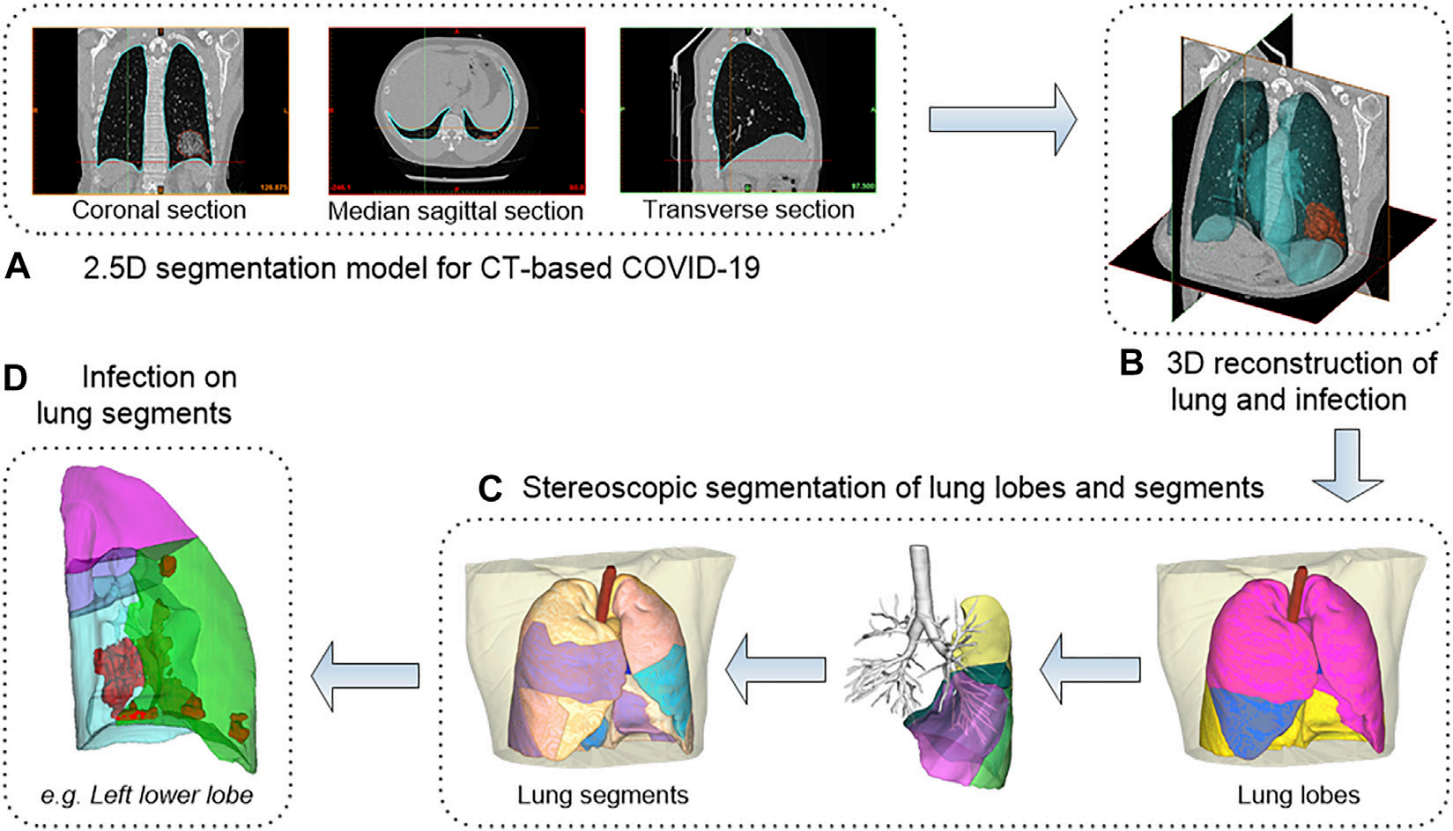
The workflow of 3D reconstruction and stereoscopic segmentation of chest CT. **(A)** shows 2.5D segmentation model for CT-based COVID-19. **(B)** shows 3D reconstruction of lung and infection. **(C)** shows stereoscopic segmentation of lung lobes and segments. **(D)** shows infection on lung segments.

Computer-aided diagnosis methods including AI lesions detection (i.e., infection segmentation) and 3D reconstruction were used to conduct an in-depth analysis of CT scans of all patients. The overall workflow is shown in Figure 4. Data included 91 CT scans from Original COVID-19 patients and 358 CT scans from Delta Variant patients. Although the CT scans of patients were collected by different devices at different hospitals with different CT thicknesses, our 2.5D segmentation model effectively overcame the notorious problem that might arise from multi-center data through the normalization method. This model was used to process all CT scans and obtained lung segmentation and infection segmentation. Segmentation results of lung and infection can be shown in the coronal section, median sagittal section, and transverse section (Figure 4A). The DICE score of this model is 0.783. The segmentation results require only a simple manual correction and can be applied clinically. Then, lung segmentation and infection segmentation were 3D reconstructed to obtain STL of lung and infection, respectively. Figure 4B shows the lung model generated after infection segmentation and 3D reconstruction of a CT scan. The lung tissue containing the pulmonary trachea was obtained by using corrosion dilatation image processing. The root of the lung trachea was cut off to achieve the separation of different lung lobes, and STL of the right upper lobe, the right middle lobe, the right lower lobe, the left upper lobe, and the left lower lobe were obtained. The centerline of the bronchus in each lobe was extracted, and the five lobes were further subdivided into 18 lung segments (Figure 4C) by isometric spherical expansion algorithm. Taking the left lower lobe as an example, it was subdivided into the dorsal segment, anterior medial basal segment, lateral basal segment, and posterior basal segment, and the STL files of each lung segment were obtained. The STL files of each lung lobe and lung segment were Boolean (intersection operation) with the STL of total infection, respectively, and finally, the distribution of infection in each lung lobe and lung segment was obtained (Figure 4D). The volume of all STL was calculated to obtain the POI values in five lung lobes and four lung segments of the left lower lobe (Table 1).

**TABLE 1:** POI values in lung segments of the left lower lobe.

	Original COVID-19	Delta Variant
Dorsal segment	0.179 (0.000, 1.551)	0.195 (0.000, 1.407)
Anterior medial basal segment	0.000 (0.000, 0.056)	0.721 (0.000, 4.264)
Lateral basal segment	0.000 (0.000, 0.323)	2.105 (0.003, 15.272)
Posterior basal segment	0.485 (0.000, 1.458)	17.872 (29.660, 67.981)

Data are medians, with ranges of quartiles in parentheses.

### 3.2 Comparative Analysis of Time Course Between the Original COVID-19 and the Delta Variant

#### 3.2.1 A New Time Course of Lung Changes in Chest CT for the COVID-19 Delta Variant

**FIGURE 5:**
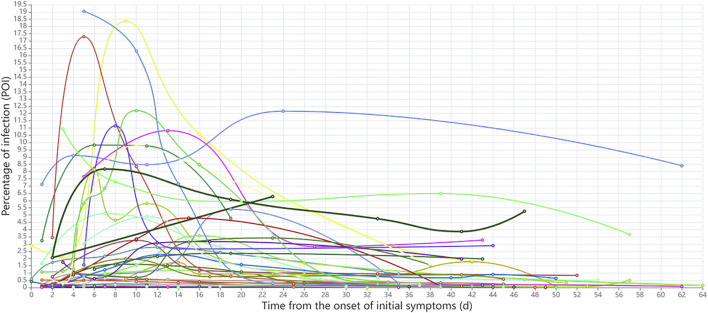
Dynamic changes of POI values since initial symptoms onset in each patient.

The dynamics of POI values for each Delta Variant patient since the initial onset of symptoms are shown in Figure 5. According to the occurrence time of peak POI value and the dynamic changes of POI, patients were initially divided into two groups: In the first group, the peak POI value appeared between 4 and 10 days, the POI value was generally larger, and the imaging manifestations were more serious. In the second group, the peak POI value appeared between 11 and 16 days, and the POI value was generally smaller, with relatively mild imaging manifestations. We found that the previously accepted and widely used COVID-19 time course is not applicable to the Delta Variant. Thus, we used the AI-based CT scoring system and nonlinear regression method to fit the scores of all the CT scans of Delta Variant patients to explore the time course of Delta Variant. In the Original COVID-19 dataset, the mean number of scans per patient was 3, and the median number of days between scans was 6. In the Delta VOC dataset, the average number of scans per patient was five and the median number of days between scans was 7. There was no significant difference in the number of CT scans and interval days between the two datasets. Because of the improved CT scoring method, we used the new scoring method to calculate the total CT scores of Original COVID-19 patients and then used the Original COVID-19 analysis results to verify the accuracy of the new scoring method.

**FIGURE 6:**
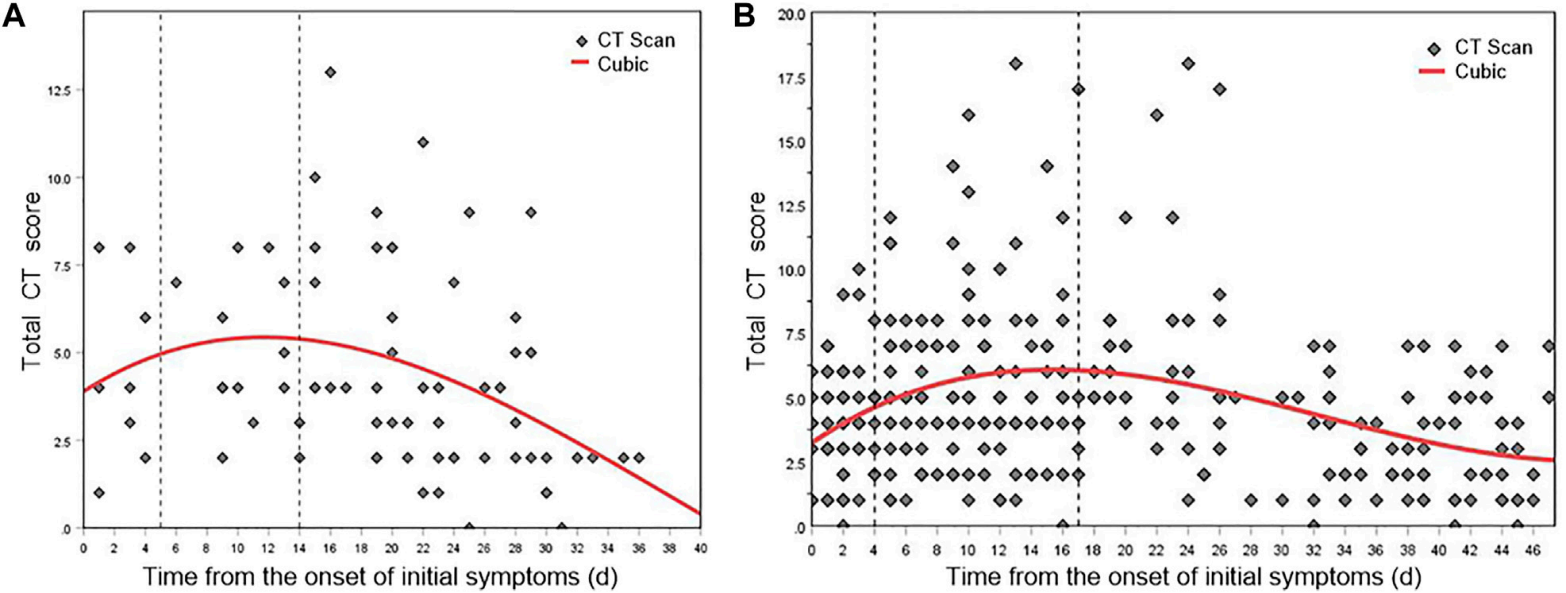
Mean dynamic trends in lung involvement since the onset of initial symptoms (quantified by total CT scores). **(A)** Total CT score distribution and fitting curve of Original COVID-19 patients. **(B)** Total CT score distribution and fitting curve of Delta Variant patients.

Figure 6A and Figure 6B show the nonlinear regression curves of lung involvement in patients with Original COVID-19 and Delta Variant, respectively. Formula (2) is the fitting equation of Original COVID-19, and Formula (3) is the fitting equation of Delta Variant.

$$y=0.0001x^{3}-0.0142x^{2}+0.2811x+3.8972$$ (2)

$$y=0.0002x^{3}-0.0178x^{2}+0.4140x+3.2357$$ (3)

where *x* represents the time from the onset of initial symptoms and *y* represents the total CT score, *p* < 0.001.

The dynamic changes of lung involvement in Original COVID-19 can be divided into three stages based on CT findings, total CT scores, and regression curve at 0–40 days after the onset of initial symptoms: stage 1 (0–4 days), stage 2 (5–13 days), and stage 3 (after 14 days). POI value increased gradually in stages 1–2 and reached the peak at day 11, and then decreased gradually in stage 3. In stage 1, CT showed the localized distribution of lesions. Symptoms such as GGO may be accompanied by the thickening of the interlobular septum. At this time, CT scores generally increase slowly. In stage 2, the lesions rapidly aggravated and extended. Bilateral lung gradually transitioned from crazy-paving pattern to consolidation and appeared “white lung” and a small amount of pleural effusion. At this time, CT scores progress rapidly. In stage 3, CT findings included that lesions decreased, density decreased, infection absorbed gradually, and residual pulmonary fibrosis. At this time, CT scores generally descend. This conclusion was consistent with the time course of Original COVID-19, which is widely accepted and applied by the academic community. It proved the accuracy of the AI-based CT scoring system. We have named stages 1–3 as early stage, progressive and peak stage, and absorption stage based on the widely used pulmonary imaging staging methods.

The dynamic changes of lung involvement in Delta Variant can also be divided into three stages based on CT findings, total CT scores, and regression curve at 0–48 days after the onset of initial symptoms: stage 1 (early stage, 0–3 days), stage 2 (progressive and peak stage, 4–16 days), and stage 3 (absorption stage, after 17 days). The peak of lung involvement occurred on day 15 of stage 2. In stage 1, most patients had multiple early lesions, a small piece of ground glass shadow can be seen under the pleura of one or both lungs, and some consolidation foci can also be seen. Thickening of interlobular septum and thickening of vascular shadow in the lesions were observed in some cases. Early CT images of a few patients showed no abnormal changes. In stage 2, the lesion range was larger than before, showing honeycomb, lump, or strip shape. Moreover, the density became denser than before, showing high-density shadow or solid change shadow. Most new lesions were also visible, and some critical patients might have diffuse changes that appear as “white lung”. It might be accompanied by thickening of vascular shadow in the lesions, bronchial inflation, and paving stone changes. Pleural or interlobular septal thickening can be spotted. In addition, some of the original lesions were more absorbed than before, but new lesions can be seen. There have been changes one after another; a few patients may have pleural effusion. In stage 3, the lesions were absorbed more than before, the range was reduced, and the density became lighter. Lesion organization and fibrosis can be observed while new lesions were seen. A few patients may have interlobular septal thickening, fibrosis and pulmonary interstitial fibrosis, and even lung volume reduction; some bronchioles may be slightly dilated, and pleural effusion was more absorbed than before.

#### 3.2.2 Comparison of Time Course of the Original COVID-19 and the Delta Variant

The time course of the Original COVID-19 and the Delta Variant is presented in Table 2. There was no significant difference between the two incubation periods. The median time from initial symptom onset to discharge from the hospital in the Original COVID-19 and the Delta Variant was 28 days (interquartile range, 21–31 days) and 42 days (interquartile range, 34–46 days), *p* < 0.001. Among them, the ranges of the early stages had no significant difference, while the range of progressive and peak stage in Delta Variant was longer than that in the Original COVID-19 (4–16 days vs. 5–13 days). The peak value of lung involvement in the Original COVID-19 and the Delta Variant was day 11 and day 15 after the onset of initial symptoms, respectively. The absorption stage of the Delta Variant occurred later and lasted longer than the Original COVID-19 (17–42 days vs. 14–28 days). The first RT-PCR negative time in Original COVID-19 patients appeared earlier than in Delta Variant patients (median [interquartile range], 22 [17–30] vs. 39 [31–44], *p* < 0.001). Delta Variant patients had more re-detectable positive RT-PCR test results than Original COVID-19 patients after the first negative RT-PCR time (30.5% vs. 17.1%).

**TABLE 2:** Different time courses of Original COVID-19 and Delta Variant.

	Original COVID-19	Delta variant
Incubation period (days before the onset of initial symptoms)	4 (2–7)	4 (3–5)
Early stage (days after the onset of initial symptoms)	0–4	0–3
Progressive and peak stage (days after the onset of initial symptoms)	5–13	4–16
Peak value of lung involvement	11	15
Absorption stage (days after the onset of initial symptoms)	14–28	17–42
Negative time of RT-PCR (days after the onset of initial symptoms)	22 (17–30)	39 (31–44)
Discharge time (days after the onset of initial symptoms)	28 (21–31)	42 (34–46)

Incubation period, negative time of RT-PCR, and discharge time are medians, with ranges of quartiles in parentheses. Incubation period is the interval between the potential earliest date of contact of the transmission source and the potential earliest date of symptom onset. Negative time of RT-PCR is the interval between the potential earliest date of symptom onset and the first negative date of RT-PCR. Discharge time is the interval between the potential earliest date of symptom onset and the discharge date.

### 3.3 Lung Differences in Patients with the Original COVID-19 and the Delta Variant

#### 3.3.1 Comparison of Dynamic Processes of Lung Involvement

According to the time course of CT scans, we compared CT scores of Delta Variant patients and Original COVID-19 patients in each lung lobe (Table 3). The changing trend of the scores of each lung lobe in three stages was first increasing and then decreasing. There was a significant difference in the CT score of the right middle lobe in the absorption stage (Delta Variant vs. Original COVID-19, 0.6 ± 0.7 vs. 0.3 ± 0.4, *p* = 0.012). There was a significant difference in the CT score of the left upper lobe in the absorption stage (Delta Variant vs. Original COVID-19, 0.9 ± 0.8 vs. 0.5 ± 0.5, *p* = 0.004). In the early stage, CT scores in the right lower lobe were significantly different (Delta Variant vs. Original COVID-19, 0.8 ± 0.6 vs. 1.3 ± 0.6, *p* = 0.039).

**TABLE 3:** CT scores of patients in each lung lobe.

		Early stage	Progressive and peak stage	Absorption stage
Right upper lobe	Delta Variant	0.5 ± 0.5 (0–2)	0.8 ± 0.6 (0–3)	0.7 ± 0.7 (0–3)
	Original COVID-19	0.8 ± 0.7 (0–1)	1.0 ± 0.4 (0–2)	0.6 ± 0.6 (0–2)
Right middle lobe	Delta Variant	0.5 ± 0.5 (0–2)	0.8 ± 0.7 (0–4)	0.6 ± 0.7 (0–4)^*^
	Original COVID-19	0.5 ± 0.5 (0–1)	0.8 ± 0.7 (0–2)	0.3 ± 0.4 (0–1)^*^
Right lower lobe	Delta Variant	0.8 ± 0.6 (0–2)^*^	1.2 ± 0.9 (0–4)	0.9 ± 0.8 (0–4)
	Original COVID-19	1.3 ± 0.6 (0–2)^*^	1.4 ± 0.5 (1–2)	0.8 ± 0.7 (0–3)
Left upper lobe	Delta Variant	0.7 ± 0.4 (0–2)	1.0 ± 0.7 (0–4)	0.9 ± 0.8 (0–4)^*^
	Original COVID-19	0.6 ± 0.5 (0–1)	0.6 ± 0.4 (0–1)	0.5 ± 0.5 (0–2)^*^
Left lower lobe	Delta Variant	1.2 ± 0.8 (0–4)	1.4 ± 0.8 (0–4)	1.2 ± 0.9 (0–4)
	Original COVID-19	1.4 ± 0.5 (1–2)	1.3 ± 0.6 (0–2)	1.1 ± 0.7 (0–4)

Data are means ± standard deviations, with ranges in parentheses.

*Represents significant difference obtained by Mann–Whitney *U* test (*p* < 0.05).

#### 3.3.2 Comparison of Prone Locations of Pneumonia Lesions

**FIGURE 7:**
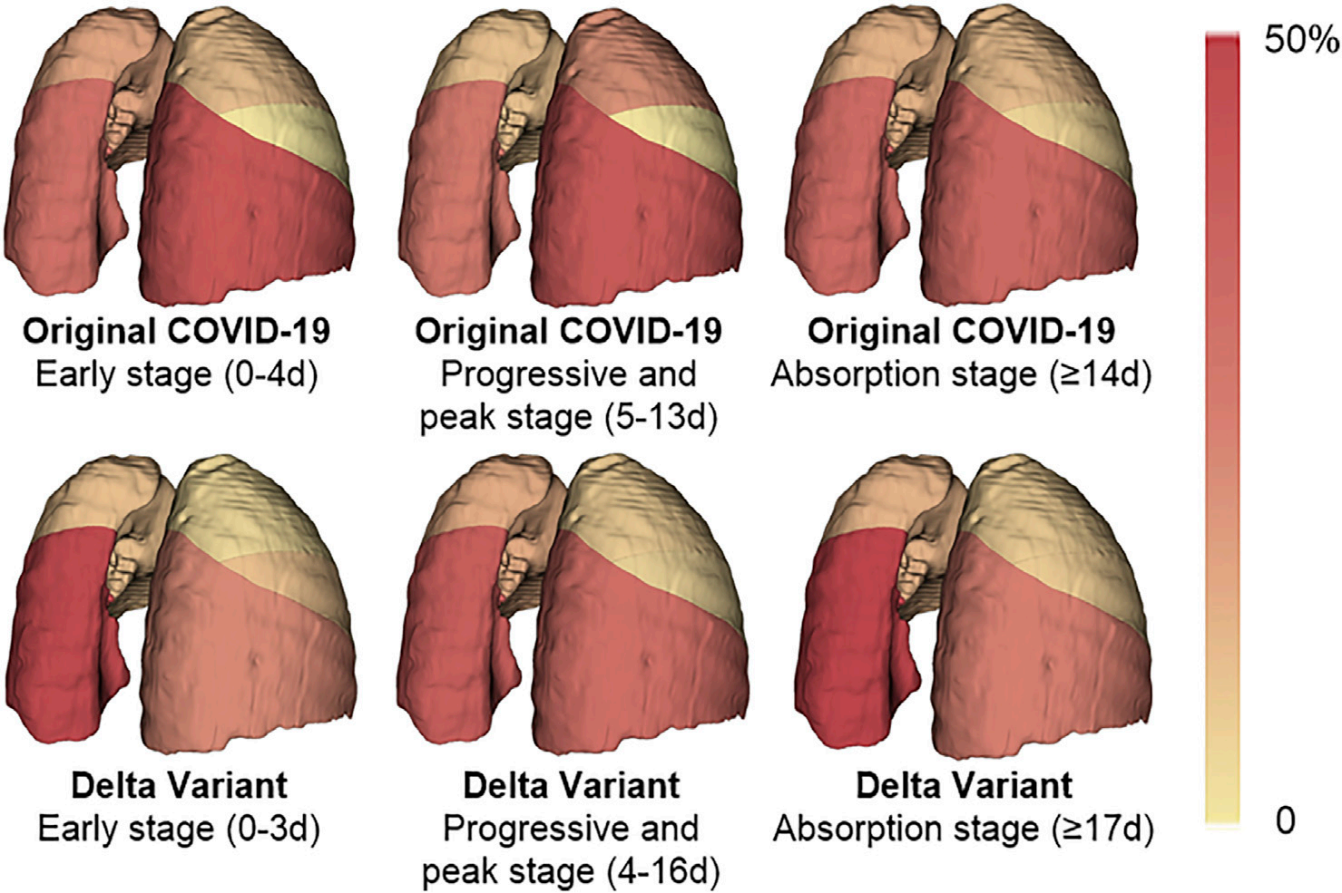
A heat map of the distribution of pneumonia. The color of the lung lobe represents the percentage of infection in the lobe to infection in the whole lung. The percentage is taken from the average of patients. The deeper the color is, the larger the percentage is.

The infection of COVID-19 patients has changed dynamically as their disease progressed and improved. We compared the lung changes of patients with the Original COVID-19 and the Delta Variant in different stages during treatment (Figure 7), and analyzed the temporal and spatial characteristics of the infection. In general, the left lower lobe and the right lower lobe were more seriously involved in different stages. In patients with the Original COVID-19, the right lower lobe contributed the most to the infection, contributing an average of 41.927% of the infection in the early stage, 40.573% in the progressive and peak stage, and 34.614% in the absorption stage. The second was the left lower lobe, which contributes 31.077%, 28.238%, and 33.768% to infection in the three stages, respectively. The total contribution of the left lower lobe and the right lower lobe in Original COVID-19 patients to infection in the whole clinical process reached 68%–73%. Among Delta Variant patients, the left lower lobe contributed the most to pneumonia, contributing 47.985% of infection in the early stage, 39.086% of infection in the progressive and peak stage, and 51.737% of infection in the absorption stage. The second is the right lower lobe, which contributes 22.863%, 32.488%, and 25.356% to infection in the three stages, respectively. The total contribution of the left lower lobe and the right lower lobe in Delta Variant patients to infection in the whole clinical process reached 71%–77%.

**FIGURE 8:**
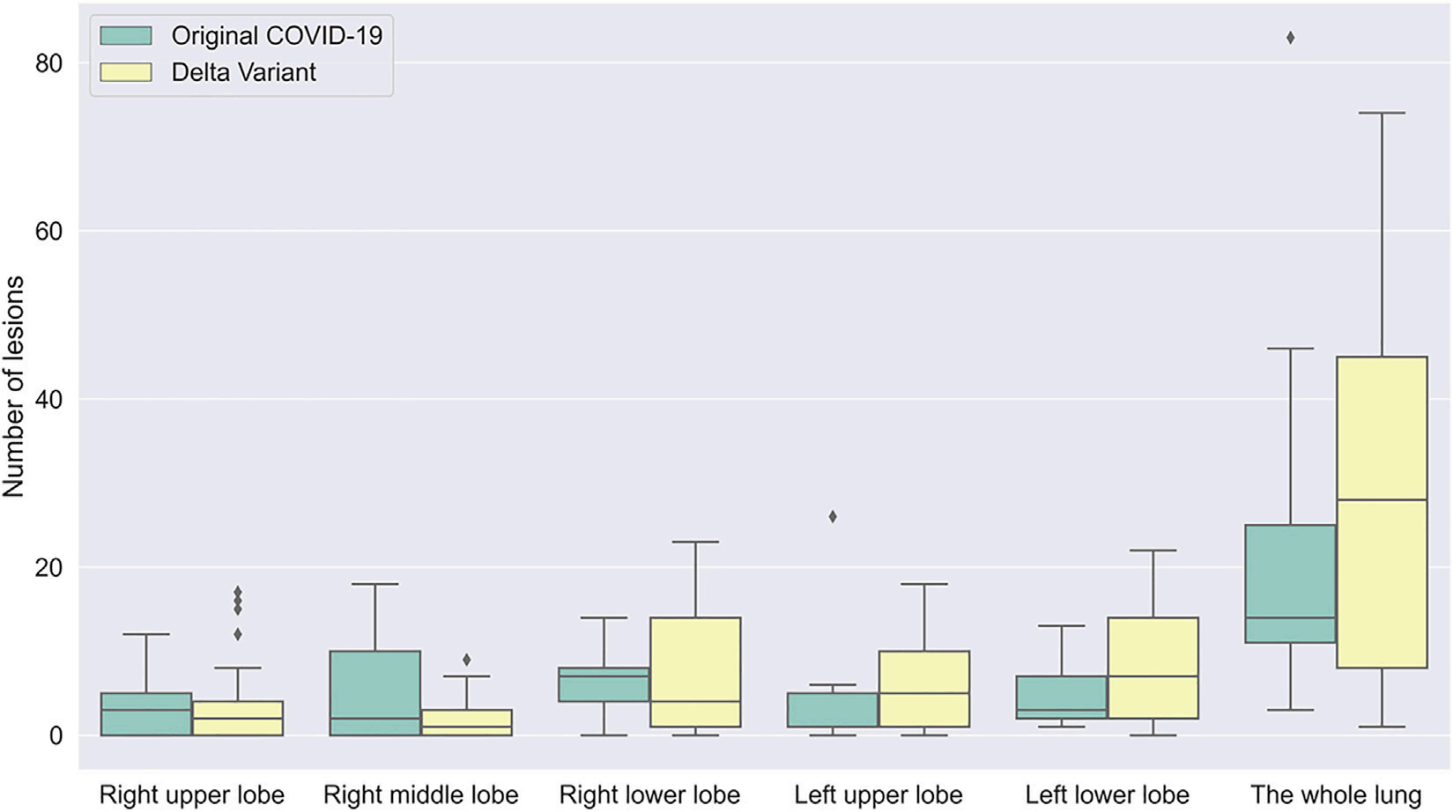
A boxplot comparing the number of lesions in early stage in patients with Original COVID-19 and Delta Variant. The abscissa indicates different regions, and the ordinate indicates the number of lesions. Early stages of Original COVID-19 and Delta Variant refer to 0–4 and 0–3 days, respectively.

According to chest CT of patients scanned in the early stage, the number and distribution of infections in patients with Original COVID-19 and Delta Variant were analyzed and compared (Figure 8). We counted the number and location of infections by the 3D reconstructed lung and infection models. Overall, both of them had infection in five lobes in the early stage. The number of infection in Delta Variant patients was significantly higher than that in Original COVID-19 patients. The number of infections in the left lower lobe and the right lower lobe was generally more than the other three lobes.

## 4 Discussion

SARS-CoV-2 continues to evolve and mutate, generating a variety of variants, and the lung imaging characteristics and clinical characteristics of infected patients are also changing continuously (Wang et al., 2021). Among them, Delta VOC has become a major variant globally since April 2021 (World Health Organization, 2021). To our knowledge, this is the first retrospective study based on chest CT comparing the similarities and differences between the Original COVID-19 and the COVID-19 Delta Variant. In this study, we used deep learning and 3D reconstruction for the COVID-19 lesion detection, 3D reconstruction of lungs and infections, and quantitative analysis of lesions in 91 CT scans of 35 Original COVID-19 patients and 358 CT scans of 72 Delta Variant patients. In addition, we proposed a computer-aided stereoscopic segmentation method, which can automatically divide the lung 3D model into five lung lobes and 18 lung segments. Using these methods, we completed segmentation and 3D reconstruction of lungs and infections on CT scans of all patients, and subdivided each lung into five lobes for accurate calculation of POI values. Combined with the POI values and clinical data, we observed that compared with the Original COVID-19 patients, Delta Variant patients have longer lung change duration, longer interval between the potential earliest date of symptom onset and the first negative date of RT-PCR, more re-detectable positive RT-PCR test results, different locations of pneumonia, and more lesions in the early stage, and the peak of infection occurred later.

RT-PCR is the gold standard for the COVID-19 diagnosis. However, affected by some factors such as sampling and detection reagents, false-negative results occur frequently (Ai et al., 2020). This confuses the diagnosis of COVID-19. Huang et al. (2020) had shown that when RT-PCR is negative, the patient’s chest CT might show early typical lung consolidation. Ai et al. (2020) confirmed that chest CT is highly sensitive to the COVID-19 diagnosis, which is an important method of screening, evaluation, and follow-up of COVID-19. In addition, traditional radiologists had to spend a lot of time reviewing CT scans of COVID-19 patients, and the long hours of high workload could result in small lesions being visually omitted. Recent studies have shown that deep learning has achieved excellent results in computer vision tasks and is close to the level of human experts in some medical image analysis tasks, such as auxiliary diagnosis of pulmonary nodules and classification of benign and malignant tumors. Therefore, we developed an online COVID-19 modeling platform based on deep learning and 3D reconstruction. It can accurately segment the regions of COVID-19 infection on CT scans and automatically generate 3D models of lungs and infections, assisting clinical diagnosis in a timely manner and effectively compensating for the high false-negative rate of RT-PCR. Based on the 3D reconstruction models, doctors can intuitively and clearly observe the distribution of infection in each lung lobe and lung segment, and evaluate the disease at the anatomical level. At present, the platform has been applied in the First Affiliated Hospital of Harbin Medical University and Zhangjiajie City People’s Hospital. We were also concerned that doctors could not quantify the lesions in the CT scans. Kazerooni et al. (Fourdrain et al., 2017) proposed a semi-quantitative scoring system to quantify the degree of involvement of each lung lobe. This scoring system has been widely used over the past 20 years, but it only mapped roughly the POI value in each lung lobe to six ranges. Our publicly available COVID-19 modeling platform enables accurate detection and quantitative analysis of COVID-19 lesions in less than 2 min, providing more objective data to assess disease severity and assist doctors in monitoring disease progression. This has greatly improved the quality of treatment. Using the stereoscopic segmentation method, the 3D model of a patient’s lung can be further subdivided into five lung lobes and 18 lung segments. This helps doctors quickly locate the infection and accurately calculate the degree of involvement of each lung lobe and lung segment as a reference to determine the clinical course of the disease.

Based on CT scans, we found that the Delta Variant had a significantly different progression of disease from the Original COVID-19. Based on the dynamic change of POI values and chest CT imaging findings, the Original COVID-19 time course of lung changes can be divided into three stages: early stage (0–4 days after the onset of initial symptoms), progressive and peak stage (5–13 days after the onset of initial symptoms), and absorption stage (14–28 days after the onset of initial symptoms). The POI value peaked at day 11. This was consistent with the widely recognized conclusion put forward by Pan et al. (Hansell et al., 2008). Using the same analysis method in the Delta Variant, the time course of lung changes can be divided into early stage (0–3 days after the onset of initial symptoms), progressive and peak stage (4–16 days after the onset of initial symptoms), and absorption stage (17–42 days after the onset of initial symptoms). The POI value peaked at day 15. This indicated that patients with the Delta Variant have a longer progressive and peak stage and a later peak of lung involvement than patients with the Original COVID-19. It was noteworthy that, according to our calculation results, the peak of lung involvement in some Delta Variant patients occurred 4–10 days after the onset of initial symptoms. This was quite different from Pan et al. (Hansell et al., 2008), who proposed that the Original COVID-19 patients generally reached the peak of lung involvement after the onset of initial symptoms on 9–13 days. The progressive and peak stage is the critical moment for COVID-19 treatment. Therefore, when treating Delta Variant patients, doctors should pay more attention to the progression of the patient’s disease, and timely detection of whether the patient has entered the peak period of lung involvement to prevent the deterioration of the disease. In addition, we compared the first RT-PCR negative time, re-detectable positive RT-PCR test rate, and discharge time between Original COVID-19 patients and Delta Variant patients. We found that patients with the Delta Variant had a longer interval between the potential earliest date of symptom onset and the first negative date of RT-PCR (median [interquartile range], 39 [31–44] vs. 22 [17–30], *p* < 0.001), a higher re-detectable positive RT-PCR test rate (30.5% vs. 17.1%), and a later discharge time than patients with the Original COVID-19 (median [interquartile range], 42 [34–46] vs. 28 [21–31], *p* < 0.001).

We also compared the temporal and spatial characteristics of the infection in Original COVID-19 patients and Delta Variant patients during hospitalization. We found that the left lower lobe and the right lower lobe contributed the most to lung involvement (approximately 70%) throughout disease progression, both in the Original COVID-19 and the Delta Variant. Among them, the right lower lobe contributed the most to lung involvement in Original COVID-19 patients, while in Delta Variant patients, the left lower lobe contributed the most. In the early stage, Delta Variant patients had significantly higher involvement in the right lower lobe than Original COVID-19 patients (0.8 ± 0.6 vs. 1.3 ± 0.6, *p* = 0.039). In the absorption stage, Delta Variant patients had significantly more involvement in the right middle lobe (0.6 ± 0.7 vs. 0.3 ± 0.4, *p* = 0.012) and the left upper lobe (0.9 ± 0.8 vs. 0.5 ± 0.5, *p* = 0.004) than Original COVID-19 patients. We also counted the number of infection in each lung lobe in the early stage and found that the average number of infection in Delta Variant patients was higher than that in Original COVID-19 patients. This indicated that patients with Delta Variant develop faster in the early stage and need timely treatment. In addition, both Original COVID-19 patients and Delta Variant patients had more lesions in the left lower lobe and the right lower lobe in the early stage than the other three lung lobes.

Our current study has several limitations. First, this is a retrospective study containing data from two independent single-center cohorts with different CT scan parameters and image quality, which may lead to unavoidable CT imaging differences. Second, the Original COVID-19 occurred in early 2020, early in the epidemic, when the response to the disease was inadequate. Over time, the Chinese government’s ability to prevent and treat COVID-19 had improved, and the COVID-19 Delta Variant occurred in mid-2021, with 41.7% of Delta Variant patients enrolled in our study having received the COVID-19 vaccine, whereas the vaccine was not available at the time of their illness in Original COVID-19 patients. These factors could not be fully considered during the experiment. Therefore, in this paper, we only studied the imaging performance of Delta VOC patients and Original COVID-19 patients, at the time they received the treatment.

## 5 Conclusion

In this study, a series of computer-aided diagnostic methods such as deep learning and 3D reconstruction were used to analyze chest CT scans in COVID-19 patients, and the percentage and number of infections were quantitatively analyzed. We proposed a stereoscopic segmentation method for lung, which can automatically subdivide a patient’s lung 3D model into five lung lobes and 18 lung segments. We found that the time course of lung changes on chest CT in Delta Variant patients was different from that in previous COVID-19 patients, and proposed a new time course of lung changes for Delta Variant patients. Based on the clinical data, POI values, and numbers of infections per lung lobe, we carefully compared the duration of disease and lung changes between the Original COVID-19 patients and the Delta Variant patients. In addition, we provided an online COVID-19 modeling platform to quickly and accurately map infected regions in CT scans of COVID-19 patients. It generates 3D models of lungs and quantifies the POI values. In summary, this study provided an in-depth comparison of the differences between the Original COVID-19 and its Delta Variant, providing additional guidance for early diagnosis and treatment of Delta Variant patients.

## Data Availability

The raw data supporting the conclusions of this article will be made available by the authors, without undue reservation.
